# Winter warming in Alaska accelerates lignin decomposition contributed by *Proteobacteria*

**DOI:** 10.1186/s40168-020-00838-5

**Published:** 2020-06-05

**Authors:** Xuanyu Tao, Jiajie Feng, Yunfeng Yang, Gangsheng Wang, Renmao Tian, Fenliang Fan, Daliang Ning, Colin T. Bates, Lauren Hale, Mengting M. Yuan, Linwei Wu, Qun Gao, Jiesi Lei, Edward A. G. Schuur, Julian Yu, Rosvel Bracho, Yiqi Luo, Konstantinos T. Konstantinidis, Eric R. Johnston, James R. Cole, C. Ryan Penton, James M. Tiedje, Jizhong Zhou

**Affiliations:** 1 Department of Microbiology and Plant Biology, University of Oklahoma, Norman, OK 73019 USA; 2 Institute for Environmental Genomics, University of Oklahoma, Norman, OK 73019 USA; 3 School of Civil Engineering and Environmental Sciences, University of Oklahoma, Norman, OK 73019 USA; 4 State Key Joint Laboratory of Environment Simulation and Pollution Control, School of Environment, Tsinghua University, Beijing, 100084 China; 5 Key Laboratory of Plant Nutrition and Fertilizer, Ministry of Agriculture, Institute of Agricultural Resources and Regional Planning, Chinese Academy of Agricultural Sciences, Beijing, 100081 China; 6 Center for Ecosystem Science and Society, Northern Arizona University, Flagstaff, AZ 86011 USA; 7 College of Integrative Sciences and Arts, Arizona State University, Mesa, AZ 85212 USA; 8 Center for Fundamental and Applied Microbiomics, The Biodesign Institute, Arizona State University, Tempe, AZ 85281 USA; 9 School of Forest Resources and Conservation, Department of Biology, University of Florida, Gainesville, FL 32611 USA; 10 School of Civil and Environmental Engineering, School of Biology, and Center for Bioinformatics and Computational Genomics, Georgia Institute of Technology, Atlanta, GA 30332 USA; 11 Center for Microbial Ecology, Michigan State University, East Lansing, MI 48824 USA; 12 Earth and Environmental Sciences, Lawrence Berkeley National Laboratory, Berkeley, CA 94720 USA

## Abstract

**Background:**

In a warmer world, microbial decomposition of previously frozen organic carbon (C) is one of the most likely positive climate feedbacks of permafrost regions to the atmosphere. However, mechanistic understanding of microbial mediation on chemically recalcitrant C instability is limited; thus, it is crucial to identify and evaluate active decomposers of chemically recalcitrant C, which is essential for predicting C-cycle feedbacks and their relative strength of influence on climate change. Using stable isotope probing of the active layer of Arctic tundra soils after depleting soil labile C through a 975-day laboratory incubation, the identity of microbial decomposers of lignin and, their responses to warming were revealed.

**Results:**

The *β*-*Proteobacteria* genus *Burkholderia* accounted for 95.1% of total abundance of potential lignin decomposers. Consistently, *Burkholderia* isolated from our tundra soils could grow with lignin as the sole C source. A 2.2 °C increase of warming considerably increased total abundance and functional capacities of all potential lignin decomposers. In addition to *Burkholderia*, *α*-*Proteobacteria* capable of lignin decomposition (e.g. *Bradyrhizobium* and *Methylobacterium* genera) were stimulated by warming by 82-fold. Those community changes collectively doubled the priming effect, i.e., decomposition of existing C after fresh C input to soil. Consequently, warming aggravates soil C instability, as verified by microbially enabled climate-C modeling.

**Conclusions:**

Our findings are alarming, which demonstrate that accelerated C decomposition under warming conditions will make tundra soils a larger biospheric C source than anticipated.

Video Abstract

## Background

Nearly half of global soil organic C is stored in the northern permafrost regions (1330–1580 Pg organic C) [1, 2]. With rapid increase of temperature occurring in higher latitudes, this large C pool becomes vulnerable to microbial decomposition [3]**.** It was shown that an increase of 2 °C would accelerate the decomposition of chemically recalcitrant C by 21%, compared with only a 10% rise for chemically labile C, suggesting that chemically recalcitrant C storage is more vulnerable to global warming [4]. Therefore, the chemically recalcitrant C pool in tundra soil may act as a source for further accumulation of atmospheric greenhouse gasses because warming significantly stimulates microbially driven degradation of vulnerable C in upland tundra ecosystem, forming a positive feedback to global climate change [3, 5].

As a complex aromatic heteropolymer in plant cell walls and litters, lignin is an important component of chemically recalcitrant C [6]. In addition to fungi [7], bacteria, such as *Streptomyces viridosporus* T7A, *Pseudomonas putida* MT-2, *Nocardia*, and *Rhodococcus jostii* RHA1 [8–11], have recently been demonstrated to be potent lignin decomposers. Given that the fungal-to-bacterial ratio was declined by warming [12] and diverse terminal electron acceptors can be utilized by bacteria during recalcitrant C decomposition, bacteria play a key role in affecting soil C stability in warmed soil [13, 14]. However, the identity of bacterial lignin decomposers in tundra soils and their responses to warming remain elusive, which prevent accurate prediction of future C fate in tundra regions.

Warmer climate leads to permafrost soil thaw in tundra regions, which releases previously frozen organic C so as to be accessible for microbial decomposition [3]. In moist acidic tundra soils, microbial community subjected to warming treatment showed a higher C-decomposing capacity [15]. Abundances of chemically recalcitrant C-decomposing genes were also increased by warming in tundra soils, which corroborated with higher ecosystem respiration [1]. Moreover, warming in tundra regions promotes plant root exudates such as organic acids, sugars, and amino acids [16], which could accelerate the chemically recalcitrant C vulnerability (termed the priming effect) [17]. Given that approximately 65% of total C in tundra soil is stored as lignin, chitin, and terpenes [1, 2], it was hypothesized that warming would aggravate tundra C instability by shifting microbial community composition, increasing decomposer abundance and the decomposing capacity of chemically recalcitrant C.

## Results and discussion

### Strong warming effects on lignin decomposers

To identify potential lignin decomposers and their responses to warming, the tundra soils, which were subjected to in situ winter warming for a 1.5-year period, in parallel with unwarmed/control soils, were collected. Since the turnover time of slow C pool of tundra soil is ~ 600 days [18], the soils were starved by laboratory incubation for 975 days to completely deplete chemically labile C reserves, a practice also recommended for helping accurate modeling of soil C kinetics [19]. Subsequently, stable isotope probing (SIP) experiments were performed to label active bacterial decomposers in warmed and control samples. Intuitively, lignin should be used. However, it is difficult to label all of C atoms of lignin owing to its complex structure. More importantly, lignin is resistant to biochemical breakdown [20]; thus, incubation time for the SIP experiment has to be extended, which inevitably causes cross-feeding. Therefore, vanillin, an intermediate of lignin decomposition widely used as a model aromatic substance to detect lignin depolymerization [20, 21], was used as the SIP substrate to identify potential ligninolytic microorganisms.

**Fig. 1:**
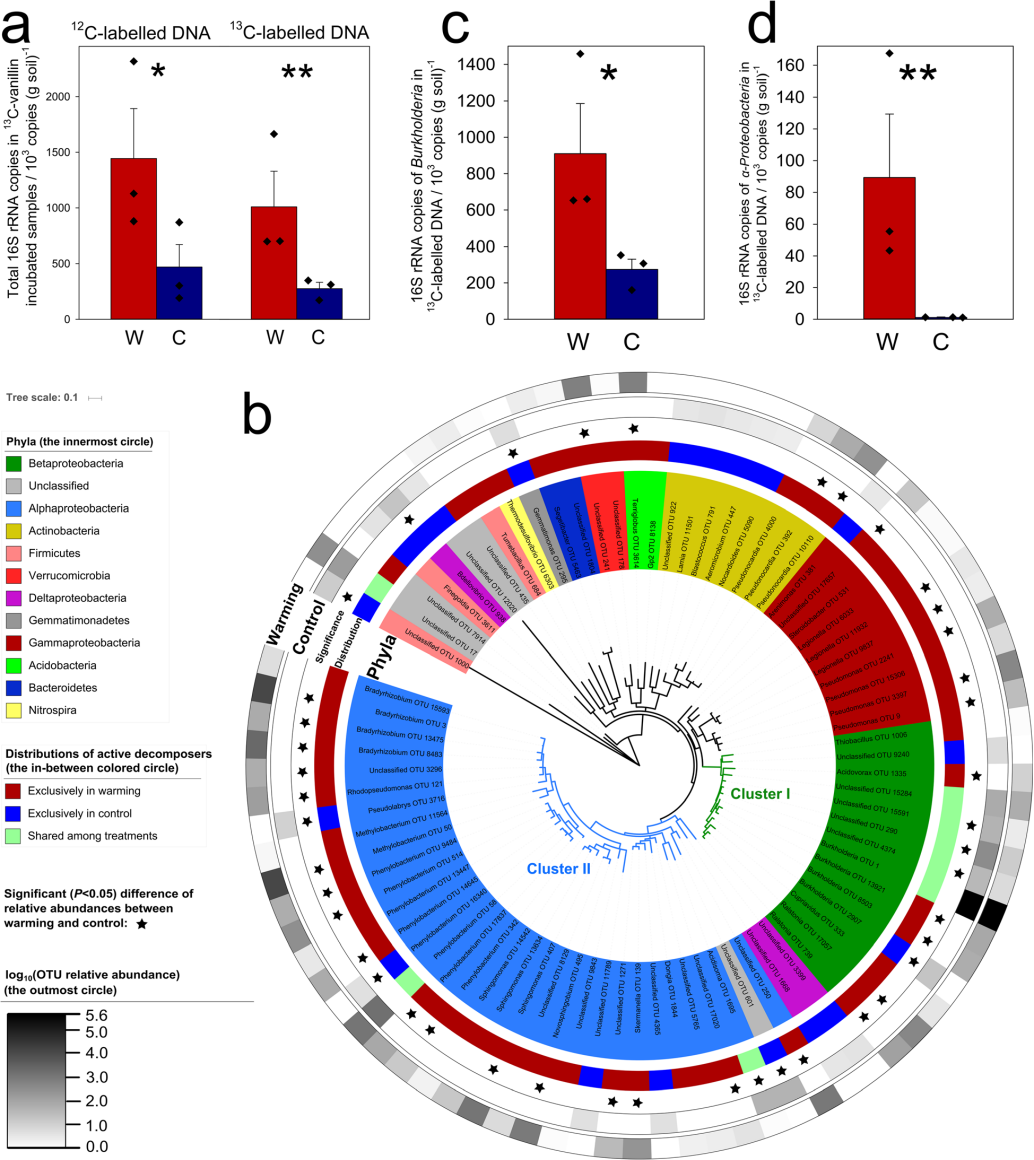
The absolute abundances of various taxa, measured by 16S rRNA genes, after incubation with ^13^C-vanillin, determined by quantitative polymerase chain reaction (qPCR) and amplicon sequencing. **a** The numbers of total ^12^C- and ^13^C-labeled 16S rRNA gene copies in ^13^C-vanillin incubated samples (*n* = 3, biological replicates of warmed or control samples). **b** The circular maximum likelihood phylogenetic tree of the ^13^C-labeled active decomposer operational taxonomic units (OTUs). Relative abundance is the modified sequence number in heavy fractions (MSNH; see the “Methods” section for details) of the OTU (*n* = 3, biological replicates of warmed or control samples), **c** The number of ^13^C-labeled 16S rRNA gene copies of *Burkholderia* (*n* = 3, biological replicates of warmed or control samples). **d** The number of ^13^C-labeled 16S rRNA gene copies of *α*-*Proteobacteria* (*n* = 3, biological replicates of warmed or control samples). *0.01 < *P ≤* 0.05 and **0.001 < *P ≤* 0.01, as determined by a two-tailed *t* test. W, warmed soils; C, unwarmed/control soils. Data are shown as mean ± standard error

The peak of ^13^C-labeled DNA was detected around the density of 1.748 g/ml (heavy fractions, Supplementary Fig. 1), which was present only in samples incubated with ^13^C-vanillin but not in samples incubated with ^12^C-vanillin or no vanillin. The peak of ^12^C-labeled DNA was detected around the density of 1.720 g/ml (light fractions). Total abundance of ^13^C-labeled DNA was increased from (2.7 ± 0.5) × 10^5^ copies/g soil in control samples to (1.0 ± 0.3) × 10^6^ copies/g soil in warmed samples (*P* < 0.01), suggesting that potential lignin decomposers were stimulated (Fig. 1a). High-throughput sequencing of ^13^C-labeled DNA showed that there were 63 operational taxonomic units (OTUs) in warmed samples, which were significantly (*P* < 0.05) higher than that of control samples (28 OTUs). In addition, warming enhanced total abundance of ^12^C-labeled DNA from (4.7 ± 1.6) × 10^5^ to (1.4 ± 0.4) × 10^6^ copies/g soil (*P* < 0.05), suggesting that growth of the overall bacterial communities was also stimulated.

Two clusters comprised of the majority of lignin decomposers were identified in a phylogenetic tree constructed from potential decomposers (Fig. 1b). Cluster I contained 14 OTUs, of which 5 OTUs were shared by both warmed and control samples. Strikingly, cluster I was solely composed of *β*-*Proteobacteria*, with 4 OTUs belonging to the genus *Burkholderia*. Although warming slightly decreased relative abundance of *Burkholderia* in ^13^C-labeled DNA from 99.4 to 90.8% (*P* < 0.01, Supplementary Fig. 2a), it increased absolute abundance of ^13^C-labeled *Burkholderia* from 2.6 × 10^5^ to 9.1 × 10^5^ copies/g soil (Fig. 1c), reflecting strong stimulation. *Burkholderiales* dominated lignocellulosic decomposers in coniferous forest soils across North America [22]. It accounted for 64% of bacterial clone sequences in Canadian High Arctic soil [23] and was identified as keystone species in Arctic heathland soils using a network analysis [24]. *Burkholderiales* are also abundant in bog ecosystems in the northern hemisphere [25, 26]. Further, relative abundance of *Burkholderia* significantly increased by 31.5-folds after the 975-day incubation (*P* < 0.05) and then increased by another 25.4-folds after addition of vanillin (*P* < 0.001), demonstrating that *Burkholderia* is highly responsive to C substrate availability (Supplementary Fig. 3). Clearly, *Burkholderia* is a keystone taxon in Arctic tundra soils (Supplementary Fig. 4).

To directly prove *Burkholderia* as lignin decomposers, two *Burkholderia* strains were isolated from tundra soils. Both strains were able to grow with lignin as the sole C source (Supplementary Fig. 5). Using whole-genome sequencing followed by genome annotation with Genome Taxonomy Database Toolkit (GTDB-tk) [27], the average nucleotide identity (ANI) between both isolate strain genomes was 99.34%, which belonged to the species of *Burkholderia zhejiangensis* [28]. A number of peroxidases were annotated in the genomes, including multiple copies of a gene encoding catalase-peroxidase well known in lignin decomposition [29]. Seven aromatic acid transporter genes and *β*-ketoadipate pathway genes associated with lignin decomposition were identified, which constitute a lignin metabolism pathway (Supplementary Fig. 6).

Cluster II contained 35 OTUs. Only two of these OTUs were shared between warmed and control samples, with 26 OTUs exclusive to warmed samples. Thirty-two cluster II OTUs belonged to *α*-*Proteobacteria*, including genera *Bradyrhizobium*, *Phenylobacterium*, *Sphingomonas*, *Novosphingobium*, and *Methylobacterium*. To provide further evidence for lignin-decomposing capacity, 7 *α*-*Proteobacterial* genomes, including 3 *Bradyrhizobium*, 3 *Phenylobacterium*, and 1 *Sphingomonas*, were assembled from deep metagenomic sequencing data of starved soils despite their low abundance compared with cluster I OTUs (Supplementary Table 1). Genes encoding catalase-peroxidases are present across all the genomes. Several genes associated with the *β*-ketoadipate pathway are also present, which are responsible for metabolizing intermediates during lignin decomposition.

Warming considerably enhanced total abundance of *α*-*Proteobacteria* in ^13^C-labeled DNA from 1.1 × 10^3^ to 8.9 × 10^4^ copies/g soil (Fig. 1d), and its relative abundance by 3.1-folds (Supplementary Fig. 2b). Among *α*-*Proteobacteria*, increases in representatives of genera *Bradyrhizobium* (from undetected to 4.0 × 10^4^ copies/g soil) and *Methylobacterium* (from undetected to 3.2 × 10^4^ copies/g soil) were most notable (Supplementary Fig. 7). Warming did not significantly change relative abundance of active *α*-*Proteobacteria* before and after the 975-day incubation at 25 °C (*P* > 0.05), but significantly stimulated it by 3.1-fold after incubation with vanillin at 25 °C (*P* < 0.05, Supplementary Fig. 8), suggesting that *α*-*Proteobacterial* decomposers were considerably induced. Typically, *α*-*Proteobacteria* prefer nutrient-rich environments and exhibit fast growth rates [30]. Tundra soil thawing by warming exposes previously frozen organic C to microbial decomposition [31], which stimulates *α*-*Proteobacteria*. Similarly, warming has increased the abundance of *α*-*Proteobacteria* in Antarctic environments [30]. However, bearing in mind a caveat that fast-growing bacteria may be preferably stimulated in the SIP experiment, slow-growing bacteria may not incorporate sufficient ^13^C-label within 6 days of incubation.

Members of *Acidobacteria* and *Actinobacteria* phyla were also identified as active lignin decomposers in coniferous forest soils across North America [22]. Consistently, two OTUs of *Acidobacteria*, which were detected only in warmed samples, and eight OTUs of *Actinobacteria* were identified as potential lignin decomposers (Fig. 1b).

### The priming effect doubled by warming

**Fig. 2:**
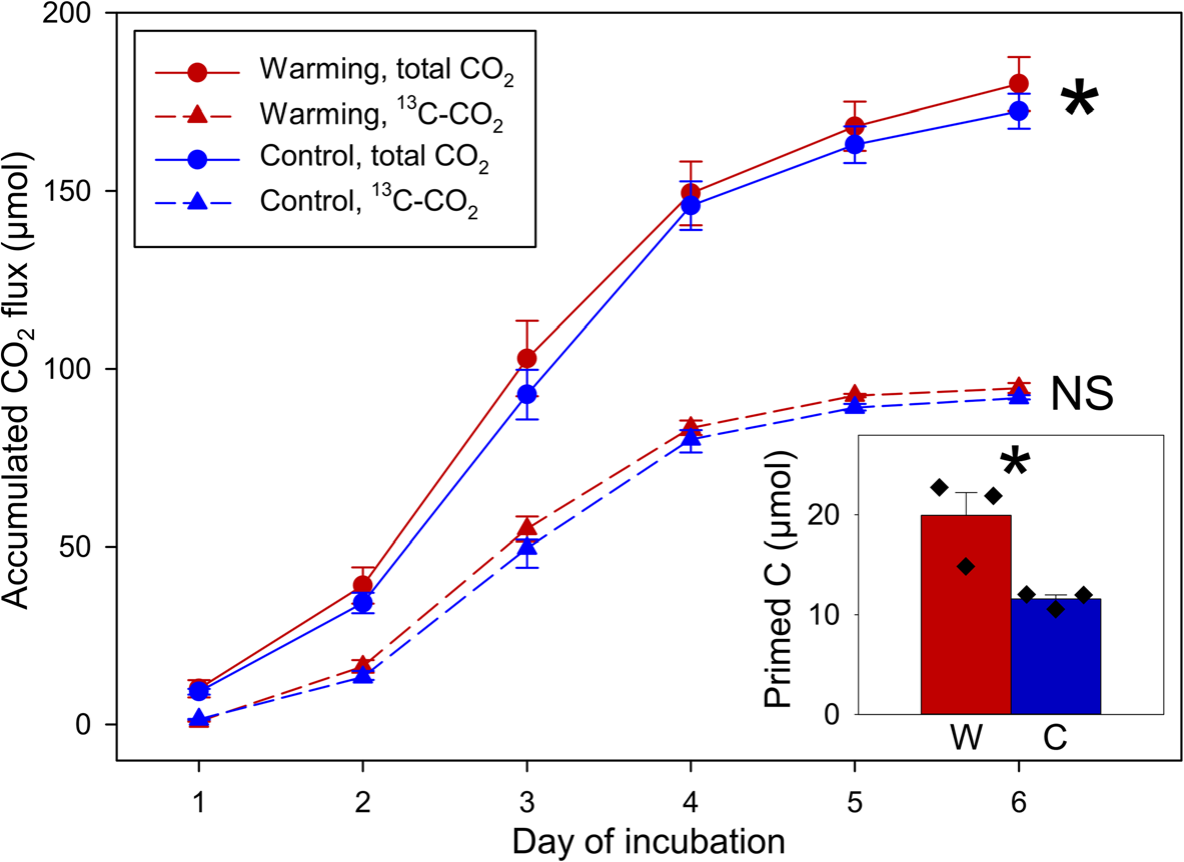
The accumulated CO_2_ flux and the priming effect during the 6-day incubation trial. Red lines/symbols represent in situ warmed soils (W) and blue lines/symbols represent unwarmed/control soils (C). Solid lines represent total CO_2_ and dashed lines represent ^13^C-CO_2_. The significance of the difference was determined by one-way ANOVA (*n* = 3, biological replicates of warmed or control samples) on CO_2_ amounts and primed C amounts between warmed and control samples (shown in the inset of the figure). W, warmed samples; C, control samples; NS, not significant. Data are shown as mean ± standard error

Changes in *α*-*Proteobacteria* abundance were positively correlated with soil CO_2_ production in the High Arctic [30]. In this study, higher abundances of *α*-*Proteobacteria* in the warmed samples (Fig. 1d) also corroborated with significantly higher total CO_2_ production measured in the SIP experiment (182.6 ± 6.2 μmol in warmed versus 174.9 ± 4.0 μmol in control samples) (Fig. 2). The ^13^C content of ^13^CO_2_ production after a 6-day incubation (92.7 ± 1.1 μmol in warmed and 90.2 ± 0.5 μmol in control samples) was close to that of added vanillin (82.6 μmol ^13^C in warmed or control samples), indicating that added vanillin was depleted by the end of the incubation period. A strong priming effect caused by supplementing with vanillin was detected, as the C molar of total CO_2_ production was substantially higher than the amount calculated from theoretical oxidation of vanillin (110.1 μmol). The C primed by vanillin under warming (19.1 ± 2.2 μmol, the inset of Fig. 2) was significantly more than that under the control condition (9.9 ± 0.5 μmol). This result contrasts a previous study that detected no significant priming effect in the organic layer of tundra soil [32]. This is likely attributed to our prolonged 975-day incubation of soils to deplete the chemically labile C reserves [31] prior to supplementing with vanillin.

**Fig. 3:**
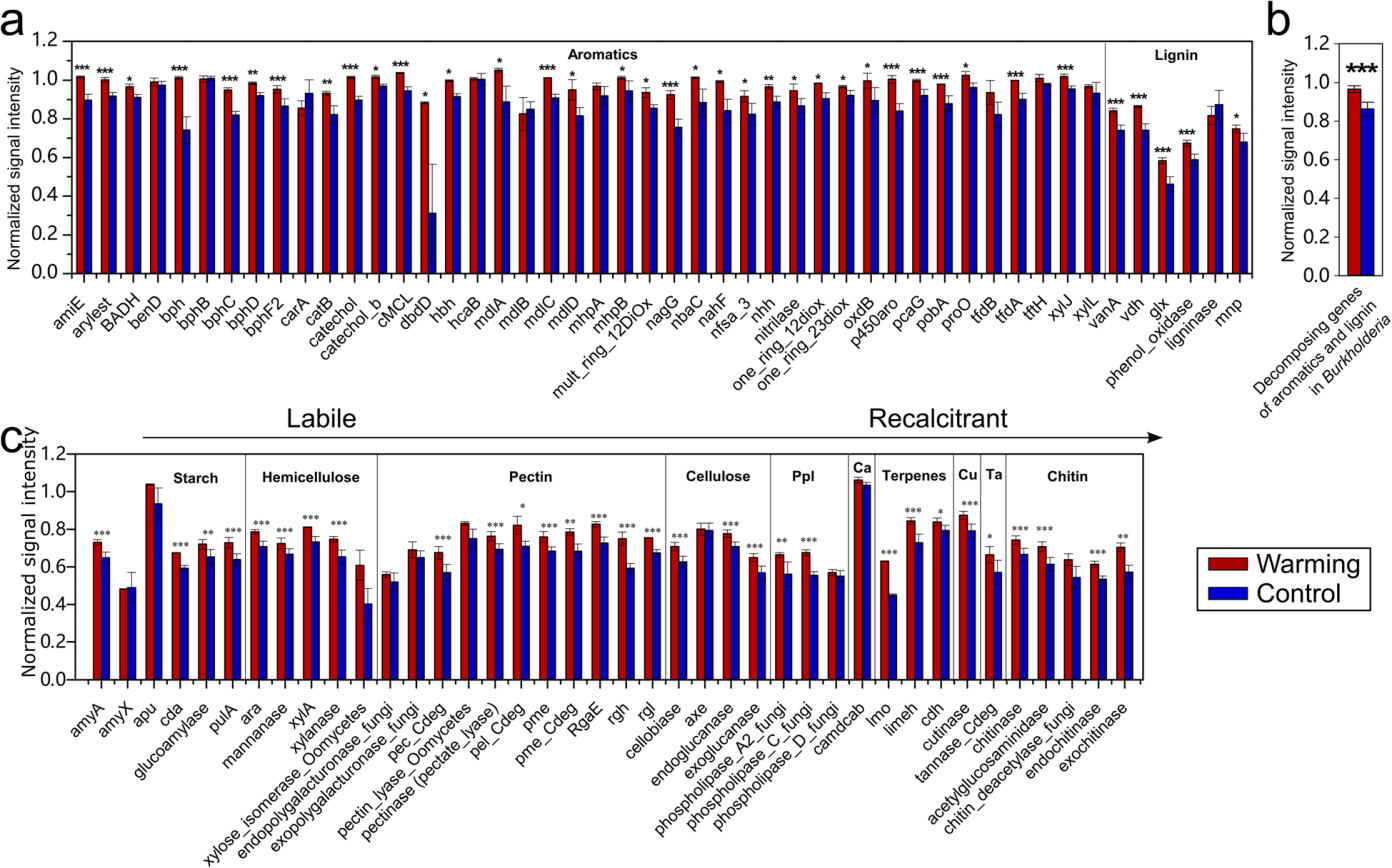
Normalized signal intensities of key C-decomposing genes in ^13^C-labeled DNA. **a** Genes associated with aromatic and lignin decomposition. **b** Aromatics- and lignin-decomposing genes belonging to *Burkholderia* in ^13^C-labeled DNA. **c** Genes associated with other key C-decomposing pathways. Ppl, phospholipids; Ca, Camphor; Cu, Cutin; and Ta, Terpenes. Differences in relative abundances were determined using one-way ANOVA (*n* = 3, biological replicates of warmed or control samples). *0.01 < *P* ≤ 0.05, **0.001 < *P* ≤ 0.01, and ***P ≤ 0.001. Data are shown as mean ± standard error

To examine whether higher CO_2_ production in warmed samples arises from changes in functional genes associated with C decomposition, especially of aromatics and lignin, relative abundances of related functional genes in ^13^C-labeled DNA were quantified by GeoChip 5.0. Strikingly, almost all detected lignin-decomposing genes, including the *mnp* gene encoding peroxidase, *lcc* genes encoding phenol oxidase, *glx* gene encoding glyoxal oxidase, *vanA* gene encoding vanillate demethylase A, and *vdh* gene encoding vanillin dehydrogenase, increased by 10.1%–26.3% under warming (Fig. 3a). This is consistent with higher ^13^C ratio and greater total CO_2_ flux under warming (Fig. 2). More broadly, 32 out of 43 aromatic-decomposing genes also significantly increased by 4.9–184.1% under warming (Fig. 3a). Among them, aromatic and lignin-decomposing genes derived from *Burkholderia* significantly increased by 11.9% (Fig. 3b), suggesting higher decomposition potentials of *Burkholderia*. Interestingly, 83.5% of the other C-decomposing genes irrelevant to lignin decomposition, including those associated with decomposing chitin (e.g., chitinase), terpenes (e.g., *cdh* encoding carveol dehydrogenase), pectin (e.g., *pel* encoding pectin lyase), cellulose (e.g., endoglucanase), hemicellulose (e.g., xylanase), and starch (e.g., *amyA* encoding α-amylase), also significantly increased under warming by 5.7–40.6% (Fig. 3c). This is likely to arise from a broad functional response of priming to warming. In contrast, no C-decomposing gene was significantly decreased in abundance by warming. Given that warming may promote plant root exudation, releasing more chemically labile C substrates to the soil [16], this strong priming effect implicates that there is an important positive feedback to global warming in tundra environments.

### Model verification and data synthesis

**Fig. 4:**
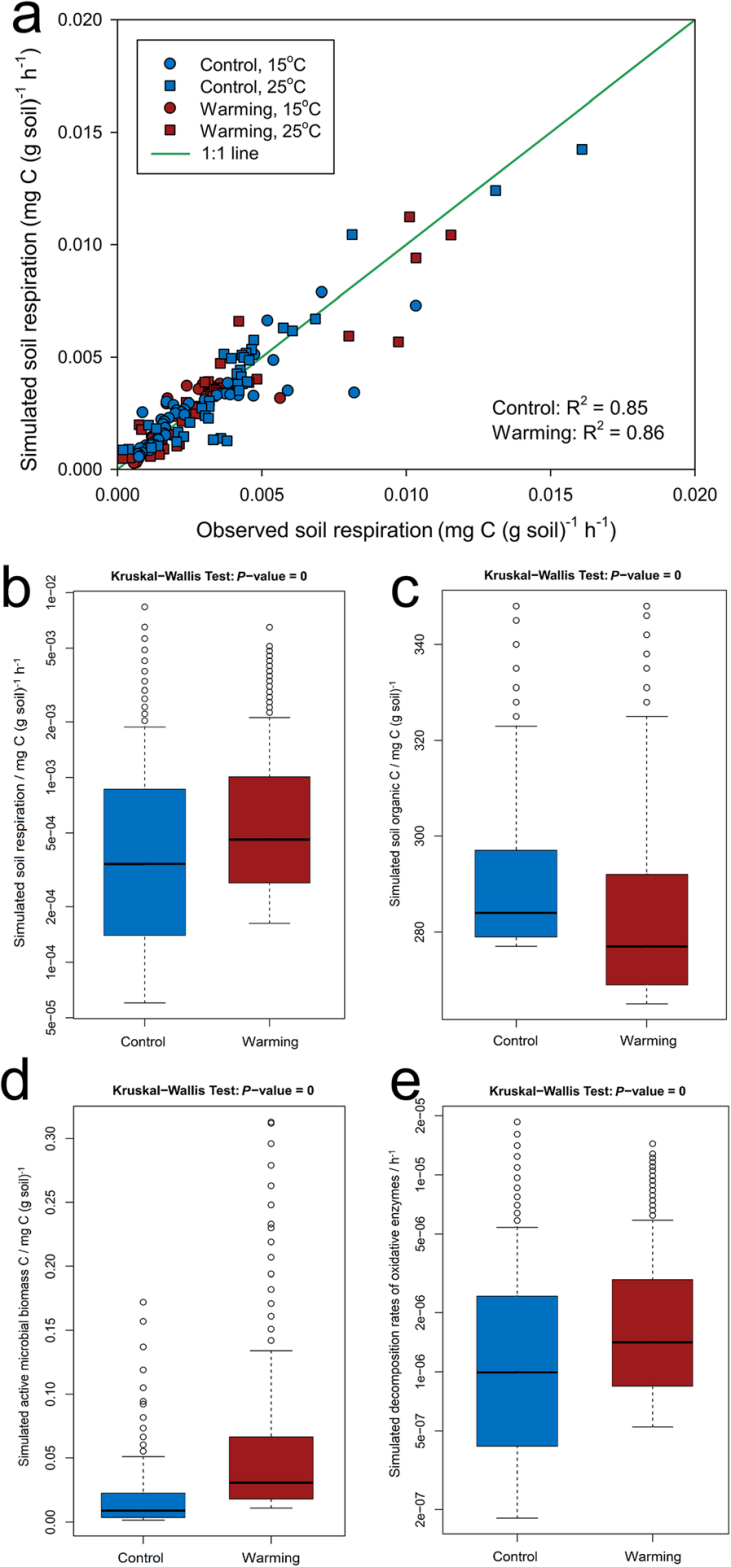
Soil and microbial variables simulated by MEND. Warming, in situ warmed soils; Control, unwarmed/control soils. **a** Simulated and observed soil respiration rates during the 975-day laboratory incubation period, showing high consistency. **b** Simulated soil heterotrophic respiration rates over 10 years. **c** Simulated soil organic C over 10 years. **d** Simulated active microbial biomass C over 10 years. **e** Simulated decomposition rates of oxidative enzymes over 10 years. The Kruskal-Wallis test was used to determine whether the parameter samples originated from significantly different distribution at a significance level of 0.05

To examine the scale of potential positive feedback of C cycling to warming, a Microbially ENabled Decomposition (MEND) model [33] was implemented to estimate the changes in C-decomposing rates. Simulated soil respiration for the 975-day laboratory incubation period agreed well with observed data (*R*^2^ ≥ 0.85, Fig. 4a). Therefore, the average soil respiration rate was simulated over the long term. The C-decomposing rates were significantly higher under warming (Fig. 4b), which were verified by in situ respiration measurements in tundra regions [1, 34]. Accordingly, tundra soil organic C is projected to decrease significantly (Fig. 4c), leading to greater net C loss under warming [1]. Higher soil respiration rates are likely caused by significantly more active microbial biomass (Fig. 4d) and higher decomposition rates of oxidative enzymes (Fig. 4e). Given that the oxidative enzymes mainly consist of ligninases [35], these modeling results are consistent with our SIP results (Fig. 1a), suggesting that warming would markedly increase the release of CO_2_ from lignin or other chemically recalcitrant C sources to the atmosphere.

## Conclusions

In this study, warming increased total abundance and functional capacities of all potential lignin decomposers in the Alaska tundra, resulting in a doubling of the priming effect on chemical recalcitrant C decomposition. Coupled with high-throughput sequencing and strain isolation, *Burkholderia* and several genera of *α*-*Proteobacteria* identified as major lignin decomposers were stimulated by warming. To our knowledge, this is the first study directly investigating active recalcitrant C-decomposing bacteria in response to warming, leading to establishment of explicit linkages between soil respiration and microbial community composition and functional capacity. The stronger priming effect under warming is alarming, which indicates a previously overlooked mechanism that accelerates climate warming in tundra regions. Given that the past 5 years are the five warmest years on record since 1880, our study provides important insights into tundra soil C stability under global warming.

## Methods

### Site description and soil sample preparation

The warming experiment was carried out at the Carbon in Permafrost Experimental Heating Research (CiPEHR) site, which was established in 2008. As described previously [3], soils were warmed during winter months by increasing snow cover behind snow fences, which were perpendicular to the dominant south-easterly winter winds. Warmed plots were on the leeward side of snow fences, while the control plots were on the windward side. Snow fences trapped and accumulated an insulating snow layer on the warmed plots. As a result, the warmed soil was at an average temperature of 2.3 °C higher than the control soil during winter.

Soil samples were collected in May 2010. Intact soil core was collected from a depth of 15–25 cm in order to avoid litter and coarse root material that constituted most of the tundra soil from the 0–15 cm layer. Soil was dried in an ED-056 oven (Sigma-Aldrich, St. Louis, MO, USA) at 60 °C until a constant weight was reached. Soil was briefly grinded before soil C and N contents were measured using an ECS 4010 Elemental Analyzer (Costech Analytical Technologies, Valencia, CA, USA). Since extended soil incubation of soil is important for identifying potential lignin decomposers and measuring the priming effect of recalcitrant C [19], intact soil samples were incubated in lightproof jars for 975 days to deplete chemically labile C. As described previously [31], soil was added to a perforated foil cup and placed over a bed of 3-mm glass beads inside the jar to allow drainage and maintain soil moisture. Because the temperature of surface soils at the CiPEHR site could reach 25 °C during the growing season [31], jars were placed in a 25 °C water bath to ensure complete depletion of chemically labile C within 975 days. Jars were covered with perforated lids to allow air exchange.

### The stable isotope probing experiment and CO_2_ flux measurement

^13^C-vanillin (vanillin-(*phenyl*-^13^C_6_); 99 atom% ^13^C) and ^12^C-vanillin were used as stable isotope probe substrates (Sigma-Aldrich, St. Louis, MO, USA). To ensure even addition to the soil, vanillin was dissolved in water as 0.8% solution, not exceeding the solubility of 1%, and then injected evenly to the soil. Three incubation groups, i.e., (1) with 0.345 ml of 0.8% ^13^C-vanillin in 2.76 g of soil (1 mg/g w/w) as isotopic treatment, (2) with 0.345 ml of 0.8% ^12^C-vanillin in 2.76 g of soil as isotopic control, and (3) with 0.345 ml of water in 2.76 g of soil as the background, were set up for both warming and control samples. Each group was set up in three biological replicates. The final water content did not exceed 70% of the water-holding capacity (WHC) of the soil, and each replicate was sealed in a 25-ml lightproof bottle and incubated at 25 °C for 6 days.

Five milliliters of headspace gas was collected daily into 12-ml evacuated vials (Labco Limited, Lampeter, UK), after which the bottle was opened and refreshed for 30 min. To generate positive pressure in relation to atmospheric pressure, sampled gas in vials were diluted by injecting 10 ml of N_2_ gas into each vial. CO_2_ and ^13^CO_2_ concentration were measured at the Stable Isotope Facility, University of California, Davis. The obtained CO_2_ and ^13^CO_2_ concentrations in parts per million were transformed to obtain the number of moles (*n*) using the ideal gas law equation *PV = nRT*, in which *P* (pressure) was 101 kPa; *V* (volume) was 25 ml multiplying the obtained CO_2_ or ^13^CO_2_ concentration in parts per million; *R* (the gas constant) was 8.314 J K^−1^ mol^−1^, and *T* (temperature) was 298 K.

The percentage of the CO_2_-C derived from vanillin was calculated as:

$$ \%{\mathrm{C}}_{\mathrm{substrate}}=\frac{\delta_{\mathrm{C}}-{\delta}_{\mathrm{T}}}{\delta_{\mathrm{C}}-{\delta}_{\mathrm{L}}}\times 100\% $$

where *δ*_C_ is the *δ*^13^C value of respired CO_2_ from the soil with no added vanillin, *δ*_T_ is the *δ*^13^C value of respired CO_2_ from the soil with ^13^C-vanillin, and *δ*_L_ is the *δ*^13^C value of ^13^C-vanillin. Because vanillin was added to the soil in the form of water solution, the amount of soil organic matter C primed by vanillin was calculated as total soil respiration after vanillin addition minus the amount of C respired from vanillin, and then minus the amount of C primed by water (C respired from the soil with no added vanillin).

### Soil DNA extraction

Soil DNA was extracted using a freeze-grinding method as described previously [36] and purified by a MOBIO PowerSoil kit (MO BIO Laboratories Inc., Carlsbad, CA, USA) according to the manufacturer’s protocol. DNA quality was assessed by a NanoDrop ND-1000 Spectrophotometer (Thermo Fisher Scientific, Waltham, MA, USA) based on spectrometry absorbance at wavelengths of 230 nm, 260 nm, and 280 nm. The absorbance ratios of 260/280 nm were larger than 1.8, and of 260/230 nm were around 1.7.

### ^13^C-DNA separation

Density gradient ultracentrifugation of ^13^C-labeled DNA was performed according to a previous protocol with minor modifications [37]. In brief, 5.1 ml of a solution was centrifuged, which was composed of 5 μg of soil DNA, 1.90 g ml^−1^ cesium chloride (CsCl) (MP Biomedicals, Santa Ana, CA, USA), and a gradient buffer of 1 mM EDTA, 0.1 M KCl, and 0.1 M Tris-HCl, reaching a final density of 1.725 g ml^−1^. The solution was sealed in a polyallomer centrifuge tube (cat. no. 342412, Beckman Coulter, Brea, CA, USA) with a cordless tube topper, and centrifuged on a Vti 65.2 rotor of an Optima L-XP ultracentrifuge (Beckman Coulter, Brea, CA, USA) at 177,000 g and 20 °C for 48 h. Solution from each centrifuged tube was then separated into twenty-four 220-ml fractions (14 drops per fraction) according to the density gradient. The buoyant density of each fraction was determined by an AR200 digital refractometer (Reichert, Depew, NY, USA). To this end, DNA in each fraction was precipitated with 20 μg of glycogen and 2 volumes of PEG solution (30% PEG 6000 and 1.6 M NaCl), washed with 70% ethanol, and re-suspended in 35 μl of ultrapure water.

Quantitative PCR (qPCR) was used to determine absolute abundances of 16S rRNA genes, thus identifying the fractions containing ^13^C-DNA. Universal primers 515F (5′-GTGCCAGCMGCCGCGGTAA-3′) and 806R (5′-GGACTACHVGGGTWTCTAAT-3′) were used for targeting the V4 region of the 16S rRNA genes. qPCR was performed in triplicate 20-μl reactions containing 10 μl SsoAdvanced™ Universal SYBR® Green Supermix (Bio-Rad, Hercules, CA, USA), 350 nM each primer and 1 μl of template, using a thermocycler program of 35 cycles of 95 °C for 20 s, 53 °C for 25 s, and 72 °C for 30 s on an IQ5 Multicolor Real-time PCR Detection System (Bio-Rad, Hercules, CA, USA). Gene copy numbers were determined by a standard curve constructed with 16S rRNA gene segment of *E*. *coli* JM109 competent cells and TA cloning vector (Promega, Madison, WI, USA). According to the buoyant density and 16S rRNA gene copy number, a light DNA peak was identified as ^12^C-labeled DNA, and a heavy DNA peak was identified as ^13^C-labeled DNA.

### 16S rRNA gene amplicon sequencing

A two-step PCR was performed prior to 16S rRNA gene sequencing [38]. In the two-step PCR, the first step of the V4 region of 16S rRNA genes was amplified by the primer 515F and 806R in triplicate 25 μl reaction containing 2.5 μl of 10 × AccuPrime PCR buffer (containing dNTPs) (Invitrogen, Grand Island, NY, USA), 1 μl of 10 μM forward and reverse primer, 2 μl of template DNA and 0.2 μl of AccuPrime High-Fidelity Taq Polymerase. The thermocycler program was as follows: 94 °C for 1 min, 10 cycles of 94 °C for 20 s, 53 °C for 25 s, and 68 °C for 45 s, and a final extension at 68 °C for 10 min. The second step of PCR also used a 25-μl reaction containing 2.5 μl of 10 × AccuPrime PCR buffer (including dNTPs), 1 μl of 10 μM 515F and 806R primer combined with the Illumina adaptor sequence, a pad and a linker of two bases, and a barcode sequences on the reverse primers, 15 μl of aliquot of the first step purified PCR product and 0.2 μl of AccuPrime High-Fidelity Taq Polymerase. The thermal cycling condition was the same as the first step except a cycle number of 20. PCR products from the second step were examined by agarose gel electrophoresis, and then triplicate PCR products were combined and quantified by Pico Green.

PCR products from each fraction were pooled at equal molarity and sequenced in the same MiSeq run. The pooled mixture was purified with a QIAquick Gel Extraction kit (Qiagen Sciences, Germantown, MD, USA) and re-quantified with Pico Green. The detailed protocol for MiSeq sequencing had been described previously [39].

The raw sequence reads were processed using an in-house pipeline built on the Galaxy platform. First, the FastQC (http://www.bioinformatics.babraham.ac.uk/projects/fastqc/) was used to evaluate the quality of raw sequence data. Second, the spiked PhiX reads were removed by demultiplexing with *E* value < 10^−5^. Third, sequences were sorted to corresponding samples according to their barcodes on the primers, which allowed for 0 mismatch. Fourth, Btrim was performed for quality trimming [40], then forward and reverse reads of the same sequence with at least 20-bp overlap and < 5% mismatch were combined by FLASH v1.2.5 program [41]. Combined sequences were removed if they contained ambiguous bases or were less than 240 bp, and then Uchime was used to remove the chimeric sequences [42]. Finally, OTUs were clustered using UPARSE at the 97% similarity level [43]. Each fraction was randomly resampled according to the absolute abundance of 16S rRNA gene based on qPCR result, with a resampling size of 29,845. OTUs were annotated through Ribosomal Database Project (RDP) classifier 2.5 with minimal 50% confidence score [44].

### Identification of potential lignin decomposers

The minor peak on the right side of the major peak was identified as ^13^C-labeled DNA from the abundance-density plot of ^13^C-incubated samples. For each sample, four fractions contributing to the major peak were termed as the “heavy fractions”, and four fractions contributing to the minor peak were termed as the “light fractions”.

Not all OTUs detected in the heavy fraction can be labeled by ^13^C due to interference of high GC content. In addition, OTUs are prone to sequencing errors. A strictly filtering method was developed to distinguish ^13^C-labeled OTUs from false positives ascribed to high GC content or sequencing errors. This filtering method included three consecutive steps: (i) To exclude OTUs of high GC content, a non-parametric *t* test was performed to determine the significance of sequence number difference assigned to each OTU found in heavy fractions between ^13^C-incubated samples and ^12^C-incubated samples. OTUs with insignificant *P* values were removed (*P* > 0.10). (ii) The relative abundances of remaining OTUs were subtracted from the relative abundances in heavy fractions of the corresponding ^12^C-incubated samples. The difference was termed as the modified sequence number in heavy fractions (MSNH). And (iii) to exclude OTUs from sequencing errors, OTUs were removed when its MSNH present in the heavy fractions was less than 20% of the total relative abundance. The remaining OTUs were regarded as potential lignin decomposers.

### Isolation, identification, and growth of *Burkholderia* isolates

*Burkholderia* AK1 and AK3 were isolated from soils after 975-day incubation by diluting nutrient broth media at 25 °C as previously described [45]. The 16S rRNA gene was amplified by universal bacterial 16S rRNA gene primers: 27F (5′-AGAGTTTGATCCTGGCTCAG-3′) and 1492R (5′-GGTTACCTTGTTACGACTT-3′). Genomic DNA of isolates was extracted by GenElute^TM^ Bacterial Genomic DNA Kit (Sigma-Aldrich, St. Louis, MO, USA) and sequenced by Illumina MiSeq platform. The ANI values were calculated by FastANI [46]. Genome Taxonomy Database Toolkit (GTDB-tk) [27] was also used for annotating taxonomy of AK1 and AK3 strains.

The BMM-defined medium [47] is comprised of 0.80 g L^−1^ NaCl, 1.0 g L^−1^ NH_4_Cl, 0.10 g L^−1^ KCl, 0.10 g L^−1^ KH_2_PO_4_, 0.80 g L^−1^ MgCl_2_·6H_2_O, 4.0 g L^−1^ CaCl_2_·2H_2_O, 10 g L^−1^ PIPES (pH = 6.5), trace minerals (160 mg/L Nitrilotriacetic acid, pH 6.5, 12.5 mg/L FeCl_2_·4H_2_O, 6.25 mg/L MnCl_2_·4H_2_O, 4.375 mg/L CoCl_2_·6H_2_O, 2.5 mg/L ZnCl_2_, 0.55 mg/L Na_2_MoO_4_·2H_2_O, 0.25 mg/L H_3_BO_3_, 1.25 mg/L NiSO_4_·6H_2_O, 0.025 mg/L CuCl_2_· 2H_2_O, 0.075 mg/L Na_2_SeO_3_, and 0.1 mg/L Na_2_WO_4_·2H_2_O), vitamins solution (0.02 mg/L Biotin, 0.02 mg/L Folic acid, 0.1 mg/L Pyridoxine HCl, 0.05 mg/L Thiamine HCl, 0.05 mg/L Riboflavin, 0.05 mg/L Nicotinic acid, 0.05 mg/L DL pantothenic acid, 0.05 mg/L p-Aminobenzoic acid, 0.05 mg/L Lipoic acid, 2 mg/L choline chloride, and 0.01 mg/L Vitamin B12), and 0.05% (m/v) alkali lignin (Sigma-Aldrich, St. Louis, MO, USA). Initially, isolated *Burkholderia* strains were inoculated into 50 ml of the BMM medium with 0.2% yeast and incubated at 28 °C with constant shaking at 180 rpm to an OD_600_ (optical density at 600 nm) of approximately 1.0. Then, 1 ml of culture was aseptically inoculated into three parallel culture flasks containing 50 ml of the BMM-defined medium. The flasks were incubated at 28 °C with constant shaking at 180 rpm for 9 days. Uninoculated medium was used as a control.

### Draft genome reconstruction

Metagenomic paired-end reads were merged using PEAR [48] (options: -p 0.001). All merged and non-merged reads were then quality-trimmed with the SolexaQA package [49] (options: -h 17). Merged and trimmed reads were assembled with IDBA-UD [50] (version 1.1.1; options: --mink 55 --maxk 107 --step 4 --min_contig 500). Contigs > 2 kb were used to calculate the mean coverage of each contig in each metagenome dataset (using megablast in BLAST + version 2.2.25; cutoff used: ≥ 90% of length of the query sequence, ≥ 98% nucleotide identity) [51]. Resulting contigs (> 2 kb) and coverage table were used with MetaBAT2 (options: --minCVSum 10) [52] to recover microbial population genomes. The quality of the resulted bins was assessed by CheckM [53]. The bins with > 80% completeness and < 1% contamination were used for further analysis. GTDB-tk [27] was also used for classification of all metagenome-assembled genomes (MAGs). The information of MAGs was provided in the Supplementary Table 1.

### Functional metagenomics analyses

Genomic annotation was performed using Automatic Genomic Analysis Pipeline (AGAP, version 1.2, an internal pipeline). Genomic annotation was conducted using PROKKA (version 1.11) [54]. First, finished genomes and draft genomes were submitted to gene calling using Prodigal (version 2.6) [54] with output of translated protein sequences, single mode and genetic code of bacteria and archaea. Then rRNA genes were predicted using Barrnap (version 0.7). Pseudogenes and coding sequences overlapping with tRNA and rRNA gene were removed by PROKKA. The 16S rRNA genes used for taxonomic classification were classified using RDP Classifier (version 2.12) [44]. Protein sequences were submitted to DIAMOND [55] search (BLASTp) against NCBI NR database (version Jan 2016) with *E* value cutoff of 1e− 5, coverage cutoff of 0.5 and maximum target number of 50. The BLASTp results were imported into MEGAN6 (Ultimate Edition, version 6.6) [56] for functional profiling with output of SEED Subsystem, KEGG, and COG categories. Exported tables of functional profiles were integrated for comparison of genomes. Genome binning (assigning the non-overlapping contigs to genomes) was performed by tetra-nucleotide frequency and verified by differential coverage binning [57].

### Experiments with GeoChip 5.0

Approximately 50 ng of DNA separated from heavy fractions in warming or control samples were amplified using a Templiphi kit (GE Healthcare, Little Chalfont, UK). The amplified DNA (2 μg) was labeled with fluorescent dye (Cy-3) dUTP using random primers and Klenow fragment of DNA polymerase I at 37 °C for 6 h, followed by heating at 95 °C for 3 min. Labeled DNA was then purified, dried in a SpeedVac at 45 °C for 45 min, and re-suspended in 43.1 μl of hybridization buffer containing 27.5 μl of 2X HI-RPM hybridization buffer, 5.5 μl of 10X CGH blocking agent, 2.4 μl of cot-1DNA, 2.2 μl of universal standard, and 5.5 μl of formamide. DNA was hybridized with GeoChip 5.0 (60 K) in a SL incubator (Shel Lab, Cornelius, OR, USA) at 67 °C for 24 h. Then, GeoChip arrays were washed and scanned by an MS 200 Microarray Scanner (Roche, Basel, Switzerland) at 532 nm and 635 nm. Raw signals from the scanning were processed by an online pipeline as previously described [58].

### Statistical and phylogenetic analyses

Differences of relative abundances between isotopic treatment and control groups were determined by Wilcoxon rank sum and signed-rank tests [59]. The two-tailed *t* test was used to determine the difference of richness of active communities between warming and control samples. All analyses above were performed in R software (version 3.3.2). Unless otherwise stated, mean values are given ± standard error of the mean, significant differences are determined by one-way ANOVA, and values of *P* ≤ 0.05 were considered significant.

The maximum likelihood phylogenetic tree was constructed based on the representative sequence for each OTU, as determined by UPARSE. MEGA 6.05 [60] was used to construct the phylogenetic tree with MUSCLE alignment, maximum likelihood method and a bootstrap value of 1000. The visual of final tree was generated by iTOL [61].

### Molecular ecological network analyses

Phylogenetic molecular ecological networks (pMENs) were constructed from the 16S rRNA gene sequencing data, using a random matrix theory (RMT)-based network approach [62]. To ensure reliability, only OTUs detected in at least 10 out of 12 samples were used for network construction. In brief, a matrix containing Pearson’s rho correlation between any pair of OTUs was generated. The threshold for network construction was automatically determined when the nearest-neighbor spacing distribution of eigenvalues transitioned from GOE to Poisson distributions. Consequently, a threshold of 0.74 was used in both warming and control sample networks. Random networks corresponding to all pMENs were constructed using the Maslov-Sneppen procedure with the same network size and average number of links to verify the system-specificity, sensitivity, and robustness of the empirical networks [63].

### Microbially enabled decomposition modeling

A Microbially ENabled Decomposition model (MEND) [33, 64] was used in this study. The MEND model is a sophisticated model with parameters representing microbial dormancy, resuscitation, and mortality. It simulates soil organic matter (SOM) decomposition in response to changes in environmental conditions such as temperature [33] by explicit microbe-mediated oxidative and hydrolytic processes. The temperature response is modeled by the Arrhenius equation characterized by the activation energy. Here, soil C pools were initialized by the measurements of soil organic C and microbial biomass C in the control and warmed samples at the beginning of the lab incubation experiments. To determine microbial parameters in soil samples, experimental data were combined at two experimental incubation temperatures (i.e., 15 °C and 25 °C) since differential temperature allows for more accurate estimate of model parameters. The stochastic shuffled complex evolution (SCE) algorithm was used [33] to determine model parameters by achieving the highest goodness-of-fit between modeled and measured CO_2_ fluxes. The microbial traits or parameters (e.g., microbial growth, maintenance, mortality, C use efficiency, and active versus dormant fractions) represent the microbial community characteristics related to soil C mineralization. Then, the model simulated with those parameters was run for 10 years to predict the long-term warming effect on SOM decomposition.

## Supplementary information

**Additional file 1: Supplementary Figure 1.** Distribution of 16S rRNA gene copy numbers along buoyant density of the samples in warmed and control samples. **Supplementary Figure 2.** The average relative abundance of (a) *Burkholderia* and (b) *Alphaproteobacteria* among ^13^C-labelled DNA. **Supplementary Figure 3.** The Napierian logarithm of relative abundances of *Burkholderia* at different time points of the experiment, as revealed by 16S rRNA gene amplicon sequencing. **Supplementary Figure 4.***Z*-*P* plot showing the distribution of OTUs based on their topological roles. **Supplementary Figure 5.** Growth curves of *Burkholderia* strains AK3 and AK1 in defined BMM medium with alkaline lignin as the sole C substrate (*n*=3). **Supplementary Figure 6.** Predicted lignin decomposition pathway in *Burkholderia* AK1 and AK3. **Supplementary Figure 7.** (a) The number of ^13^C-labelled 16S rRNA gene copies of *Bradyrhizobium*; (b) The number of ^13^C-labelled 16S rRNA gene copies of *Methylobacterium*. **Supplementary Figure 8.** Relative abundances of active α-Proteobacteria at different time points during the experiment, as revealed by 16S rRNA gene amplicon sequencing.

**Additional file 2: Supplementary Table 1.** Information of α-*Proteobacterial* metagenome-assembled genomes (MAGs).

## Data Availability

Raw sequences of 16S rRNA gene amplicons after 6-day incubation with vanillin are available in NCBI SRA database (http://www.ncbi.nlm.nih.gov/sra) under accession number PRJNA521395. The shotgun metagenome datasets are available in the European Nucleotide Archive under project number PRJEB31279. The genome sequences of the isolates and the metagenome-assembled genomes are available in NCBI database WGS under the project PRJNA521345. GeoChip raw and normalized signal intensities can be accessed through the URL http://129.15.40.254/NewIEGWebsiteFiles/publications/SupplData/TaoX-WarmExacDecompSOC-Nature-RawData.txt and http://129.15.40.254/NewIEGWebsiteFiles/publications/SupplData/TaoX-WarmExacDecompSOC-Nature-NormData.txt The MEND model code and data are accessible upon request at https://wanggangsheng@bitbucket.org/wanggangsheng/mend_mult.

## References

[1] Xue K, Yuan MM, Shi ZJ, Qin Y, Deng Y, Cheng L (2016). Tundra soil carbon is vulnerable to rapid microbial decomposition under climate warming. Nat Clim Chang.

[2] Vonk JE, Sánchez-García L, Van Dongen B, Alling V, Kosmach D, Charkin A (2012). Activation of old carbon by erosion of coastal and subsea permafrost in Arctic Siberia. Nature..

[3] Natali SM, Schuur EA, Trucco C, Hicks Pries CE, Crummer KG, Baron Lopez AF (2011). Effects of experimental warming of air, soil and permafrost on carbon balance in Alaskan tundra. Glob Chang Biol.

[4] Davidson EA, Janssens IA (2006). Temperature sensitivity of soil carbon decomposition and feedbacks to climate change. Nature..

[5] Schuur EA, Vogel JG, Crummer KG, Lee H, Sickman JO, Osterkamp T (2009). The effect of permafrost thaw on old carbon release and net carbon exchange from tundra. Nature..

[6] Romero-Olivares AL, Allison SD, Treseder KK (2017). Decomposition of recalcitrant carbon under experimental warming in boreal forest. PLoS One.

[7] Bugg TD, Ahmad M, Hardiman EM, Singh R (2011). The emerging role for bacteria in lignin degradation and bio-product formation. Curr Opin Biotechnol.

[8] Vicuña R (1988). Bacterial degradation of lignin. Enzym Microb Technol.

[9] Ramachandra M, Crawford DL, Hertel G (1988). Characterization of an extracellular lignin peroxidase of the lignocellulolytic actinomycete Streptomyces viridosporus. Appl Environ Microbiol.

[10] Masai E, Katayama Y, Fukuda M (2007). Genetic and biochemical investigations on bacterial catabolic pathways for lignin-derived aromatic compounds. Biosci Biotechnol Biochem.

[11] Zimmermann W (1990). Degradation of lignin by bacteria. J Biotechnol.

[12] Sistla SA, Moore JC, Simpson RT, Gough L, Shaver GR, Schimel JP (2013). Long-term warming restructures Arctic tundra without changing net soil carbon storage. Nature..

[13] DeAngelis KM, Pold G, Topçuoğlu BD, van Diepen LT, Varney RM, Blanchard JL (2015). Long-term forest soil warming alters microbial communities in temperate forest soils. Front Microbiol.

[14] Pold G, Melillo JM, DeAngelis KM (2015). Two decades of warming increases diversity of a potentially lignolytic bacterial community. Front Microbiol.

[15] Ernakovich JG, Wallenstein MD (2015). Permafrost microbial community traits and functional diversity indicate low activity at in situ thaw temperatures. Soil Biol Biochem.

[16] Yin H, Li Y, Xiao J, Xu Z, Cheng X, Liu Q (2013). Enhanced root exudation stimulates soil nitrogen transformations in a subalpine coniferous forest under experimental warming. Glob Chang Biol.

[17] Mau RL, Dijkstra P, Schwartz E, Koch BJ, Hungate BA (2018). Warming induced changes in soil carbon and nitrogen influence priming responses in four ecosystems. Appl Soil Ecol.

[18] Hale L, Feng W, Yin H, Guo X, Zhou X, Bracho R, et al. Tundra microbial community taxa and traits predict decomposition parameters of stable, old soil organic carbon. ISME J. 2019:1–15. 10.1038/s41396-019-0485-x PMC6863828 31384013

[19] Schädel C, Schuur EA, Bracho R, Elberling B, Knoblauch C, Lee H (2014). Circumpolar assessment of permafrost C quality and its vulnerability over time using long-term incubation data. Glob Chang Biol.

[20] Taylor CR, Hardiman E, Ahmad M, Sainsbury P, Norris P, Bugg T (2012). Isolation of bacterial strains able to metabolize lignin from screening of environmental samples. J Appl Microbiol.

[21] Zak DR, Kling GW (2006). Microbial community composition and function across an arctic tundra landscape. Ecology..

[22] Wilhelm RC, Singh R, Eltis LD, Mohn WW (2019). Bacterial contributions to delignification and lignocellulose degradation in forest soils with metagenomic and quantitative stable isotope probing. ISME J.

[23] Harding T, Jungblut AD, Lovejoy C, Vincent WF (2011). Microbes in high Arctic snow and implications for the cold biosphere. Appl Environ Microbiol.

[24] Hill R, Saetnan ER, Scullion J, Gwynn-Jones D, Ostle N, Edwards A (2016). Temporal and spatial influences incur reconfiguration of Arctic heathland soil bacterial community structure. Environ Microbiol.

[25] Bragina A, Berg C, Berg G (2015). The core microbiome bonds the Alpine bog vegetation to a transkingdom metacommunity. Mol Ecol.

[26] Bragina A, Cardinale M, Berg C, Berg G (2013). Vertical transmission explains the specific Burkholderia pattern in sphagnum mosses at multi-geographic scale. Front Microbiol.

[27] Parks DH, Chuvochina M, Waite DW, Rinke C, Skarshewski A, Chaumeil P-A (2018). A standardized bacterial taxonomy based on genome phylogeny substantially revises the tree of life. Nat Biotechnol.

[28] Lu P, Zheng L-Q, Sun J-J, Liu H-M, Li S-P, Hong Q (2012). Burkholderia zhejiangensis sp. nov., a methyl-parathion-degrading bacterium isolated from a wastewater-treatment system. Int J Syst Evol Microbiol.

[29] Brown ME, Walker MC, Nakashige TG, Iavarone AT, Chang MC (2011). Discovery and characterization of heme enzymes from unsequenced bacteria: application to microbial lignin degradation. J Am Chem Soc.

[30] Yergeau E, Bokhorst S, Kang S, Zhou J, Greer CW, Aerts R (2012). Shifts in soil microorganisms in response to warming are consistent across a range of Antarctic environments. ISME J.

[31] Bracho R, Natali S, Pegoraro E, Crummer KG, Schädel C, Celis G (2016). Temperature sensitivity of organic matter decomposition of permafrost-region soils during laboratory incubations. Soil Biol Biochem.

[32] De Baets S, Van de Weg M, Lewis R, Steinberg N, Meersmans J, Quine T (2016). Investigating the controls on soil organic matter decomposition in tussock tundra soil and permafrost after fire. Soil Biol Biochem.

[33] Wang G, Jagadamma S, Mayes MA, Schadt CW, Steinweg JM, Gu L (2015). Microbial dormancy improves development and experimental validation of ecosystem model. ISME J.

[34] Pries CEH, Schuur EA, Natali SM, Crummer KG (2016). Old soil carbon losses increase with ecosystem respiration in experimentally thawed tundra. Nat Clim Chang.

[35] Wang G, Post WM, Mayes MA, Frerichs JT, Sindhu J (2012). Parameter estimation for models of ligninolytic and cellulolytic enzyme kinetics. Soil Biol Biochem.

[36] Zhou JZ, Bruns MA, Tiedje JM (1996). DNA recovery from soils of diverse composition. Appl Environ Microbiol.

[37] Neufeld JD, Vohra J, Dumont MG, Lueders T, Manefield M, Friedrich MW (2007). DNA stable-isotope probing. Nat Protoc.

[38] Wu L, Wen C, Qin Y, Yin H, Tu Q, Van Nostrand JD (2015). Phasing amplicon sequencing on Illumina Miseq for robust environmental microbial community analysis. BMC Microbiol.

[39] Zhou J, Deng Y, Shen L, Wen C, Yan Q, Ning D (2016). Temperature mediates continental-scale diversity of microbes in forest soils. Nat Commun.

[40] Kong Y (2011). Btrim: a fast, lightweight adapter and quality trimming program for next-generation sequencing technologies. Genomics..

[41] Magoč T, Salzberg SL (2011). FLASH: fast length adjustment of short reads to improve genome assemblies. Bioinformatics..

[42] Edgar RC, Haas BJ, Clemente JC, Quince C, Knight R (2011). UCHIME improves sensitivity and speed of chimera detection. Bioinformatics..

[43] Edgar RC (2013). UPARSE: highly accurate OTU sequences from microbial amplicon reads. Nat Methods.

[44] Wang Q, Garrity GM, Tiedje JM, Cole JR (2007). Naive Bayesian classifier for rapid assignment of rRNA sequences into the new bacterial taxonomy. Appl Environ Microbiol.

[45] Janssen PH, Yates PS, Grinton BE, Taylor PM, Sait M (2002). Improved culturability of soil bacteria and isolation in pure culture of novel members of the divisions Acidobacteria, Actinobacteria, Proteobacteria, and Verrucomicrobia. Appl Environ Microbiol.

[46] Jain C, Rodriguez-R LM, Phillippy AM, Konstantinidis KT, Aluru S (2018). High throughput ANI analysis of 90 K prokaryotic genomes reveals clear species boundaries. Nat Commun.

[47] Woo HL, Hazen TC, Simmons BA, DeAngelis KM (2014). Enzyme activities of aerobic lignocellulolytic bacteria isolated from wet tropical forest soils. Syst Appl Microbiol.

[48] Zhang J, Kobert K, Flouri T, Stamatakis A (2013). PEAR: a fast and accurate Illumina paired-end reAd mergeR. Bioinformatics..

[49] Cox MP, Peterson DA, Biggs PJ (2010). SolexaQA: at-a-glance quality assessment of Illumina second-generation sequencing data. BMC Bioinformatics.

[50] Peng Y, Leung HC, Yiu S-M, Chin FY (2012). IDBA-UD: a de novo assembler for single-cell and metagenomic sequencing data with highly uneven depth. Bioinformatics..

[51] Camacho C, Coulouris G, Avagyan V, Ma N, Papadopoulos J, Bealer K (2009). BLAST+: architecture and applications. BMC Bioinformatics..

[52] Kang DD, Froula J, Egan R, Wang Z (2015). MetaBAT, an efficient tool for accurately reconstructing single genomes from complex microbial communities. PeerJ..

[53] Parks DH, Imelfort M, Skennerton CT, Hugenholtz P, Tyson GW (2015). CheckM: assessing the quality of microbial genomes recovered from isolates, single cells, and metagenomes. Genome Res.

[54] Hyatt D, Chen G-L, LoCascio PF, Land ML, Larimer FW, Hauser LJ (2010). Prodigal: prokaryotic gene recognition and translation initiation site identification. BMC Bioinformatics.

[55] Buchfink B, Xie C, Huson DH (2015). Fast and sensitive protein alignment using DIAMOND. Nat Methods.

[56] Huson DH, Auch AF, Qi J, Schuster SC (2007). MEGAN analysis of metagenomic data. Genome Res.

[57] Albertsen M, Hugenholtz P, Skarshewski A, Nielsen KL, Tyson GW, Nielsen PH (2013). Genome sequences of rare, uncultured bacteria obtained by differential coverage binning of multiple metagenomes. Nat Biotechnol.

[58] Yang Y, Wu L, Lin Q, Yuan M, Xu D, Yu H (2013). Responses of the functional structure of soil microbial community to livestock grazing in the Tibetan alpine grassland. Glob Chang Biol.

[59] Hollander M. DA Wolfe. 1973. Nonparametric statistical methods. John Wiley and Sons Perry, P and S Wolff.1074:156-158.

[60] Hall BG (2013). Building phylogenetic trees from molecular data with MEGA. Mol Biol Evol.

[61] Life ITO (2011). v2: online annotation and display of phylogenetic trees made easy Letunic, Ivica; Bork. Peer Nucleic Acids Res.

[62] Deng Y, Jiang Y-H, Yang Y, He Z, Luo F, Zhou J (2012). Molecular ecological network analyses. BMC Bioinformatics..

[63] Maslov S, Sneppen K (2002). Specificity and stability in topology of protein networks. Science..

[64] Wang K, Peng C, Zhu Q, Zhou X, Wang M, Zhang K (2017). Modeling global soil carbon and soil microbial carbon by integrating microbial processes into the ecosystem process model TRIPLEX-GHG. J Adv Model Earth Sys.

