# Supplementary material for: Winter warming in Alaska accelerates lignin decomposition contributed by Proteobacteria
Source: Microbiome. 2020 Jun 5;8:84. doi: 10.1186/s40168-020-00838-5 (PMC7275452; doi:10.1186/s40168-020-00838-5)
Supplement: Supplementary file 2 — Additional file 1: Supplementary Figure 1. Distribution of 16S rRNA gene copy numbers along buoyant density of the samples in warmed and control samples. Supplementary Figure 2. The average relative abundance of (a) Burkholderia and (b) Alphaproteobacteria among 13C-labelled DNA. Supplementary Figure 3. The Napierian logarithm of relative abundances of Burkholderia at different time points of the experiment, as revealed by 16S rRNA gene amplicon sequencing. Supplementary Figure 4.Z-P plot showing the distribution of OTUs based on their topological roles. Supplementary Figure 5. Growth curves of Burkholderia strains AK3 and AK1 in defined BMM medium with alkaline lignin as the sole C substrate (n=3). Supplementary Figure 6. Predicted lignin decomposition pathway in Burkholderia AK1 and AK3. Supplementary Figure 7. (a) The number of 13C-labelled 16S rRNA gene copies of Bradyrhizobium; (b) The number of 13C-labelled 16S rRNA gene copies of Methylobacterium. Supplementary Figure 8. Relative abundances of active α-Proteobacteria at different time points during the experiment, as revealed by 16S rRNA gene amplicon sequencing. [file 40168_2020_838_MOESM1_ESM.docx]

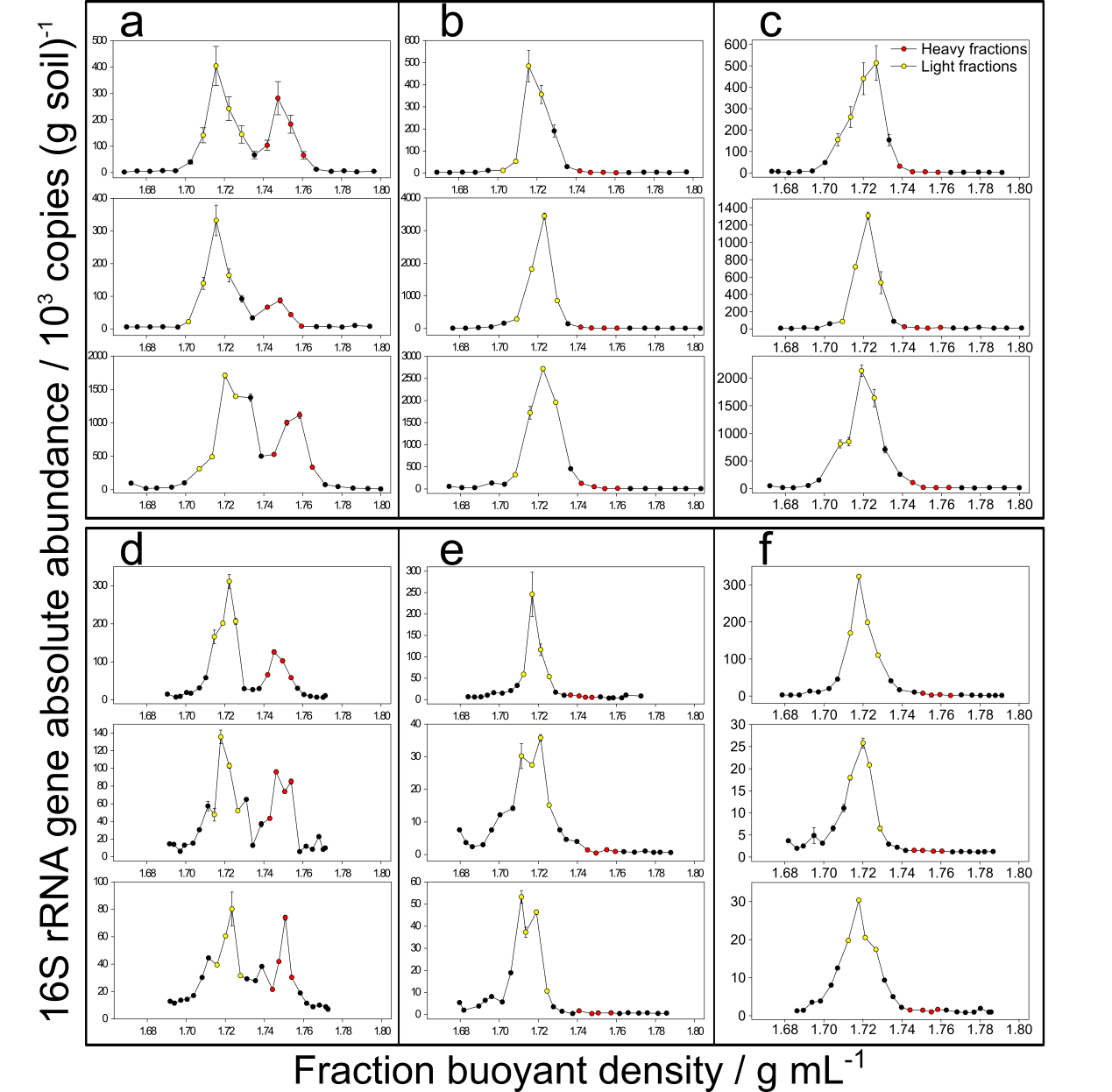


**Supplementary Figure 1.** Detection of ^13^C-labelled DNA in density gradient fractions using qPCR targeting 16S rRNA genes. The gradient fractions were derived from DNA samples of soils including: a, warmed soil with ^13^C-vanillin (isotopic treatment group); b, warmed soil with ^12^C-vanillin (isotopic control group); c, warmed soil with water (background group); d, control soil with ^13^C-vanillin; e, control soil with ^12^C-vanillin; f, control soil with water. Each group contains three biological replicates. Red symbols represent the heavy fractions (~1.748 g/ml) and yellow symbols represent the light fractions (~ 1.720 g/ml) for each biological replicate. Each biological replicate value is shown as the average ± standard error of three technical replicates.


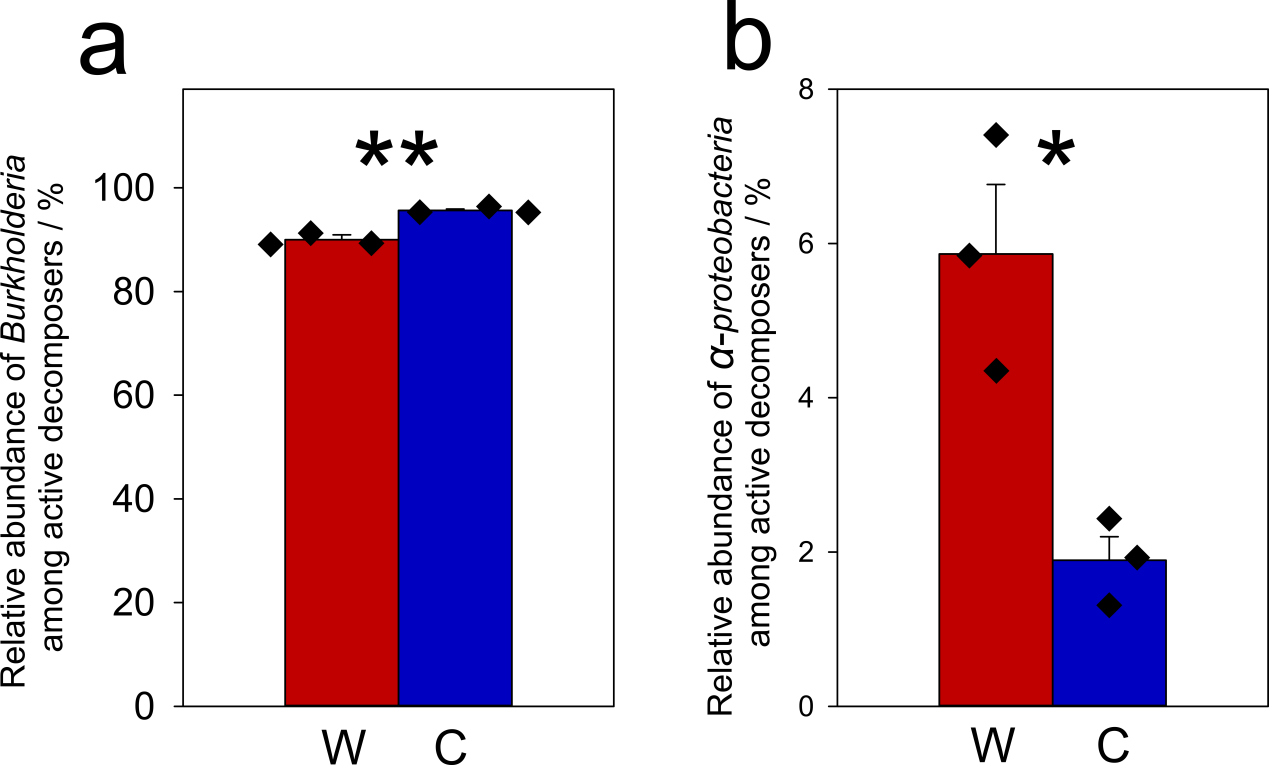


**Supplementary Figure 2.** The average relative abundance of (a) *Burkholderia* and (b) *Alphaproteobacteria* among ^13^C-labelled DNA. Significance is indicated by *, 0.01<*P*≤0.05; **, 0.001<*P*≤0.01; ***, *P*≤0.001 as determined by two-tailed *t*-test (*n*=3 biological replicates). W: warmed sample; C: control samples. Data are shown as mean ± standard error.


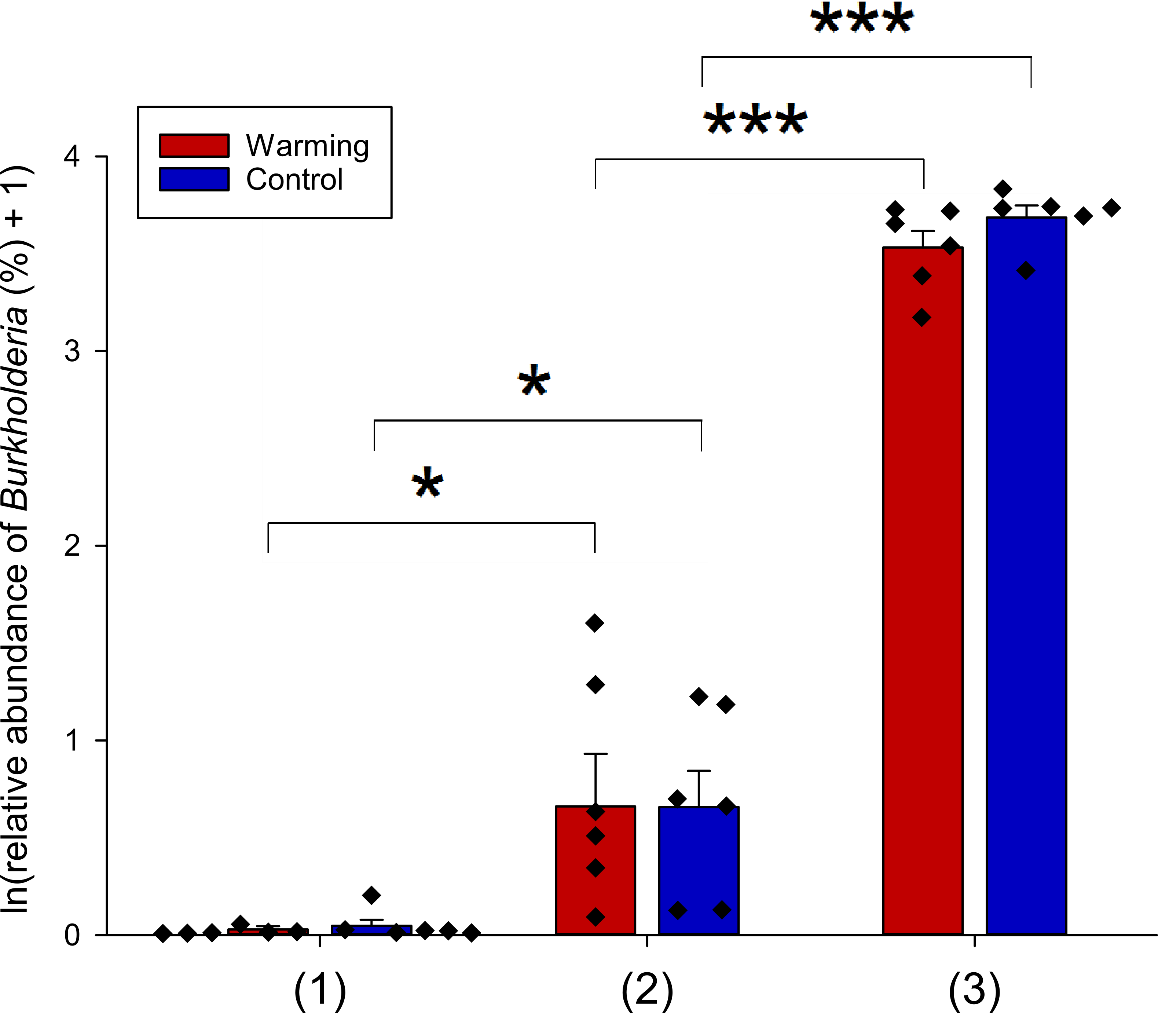


**Supplementary Figure 3.** The Napierian logarithm of relative abundances of *Burkholderia* at different time points of the experiment, as revealed by 16S rRNA gene amplicon sequencing. The X axis shows samples of different time points of (1) before the 975-day lab incubation, (2) after the 975-day lab incubation, and (3) after the 6-day SIP incubation. Significance is indicated by *, 0.01<*P*≤0.05; **, 0.001<*P*≤0.01; ***, *P*≤0.001, as determined by two-tailed *t*-test (*n*=6 before and after 975-day laboratory incubation, and *n*=3 after the 6-day SIP incubation). Data are shown as mean ± standard error.


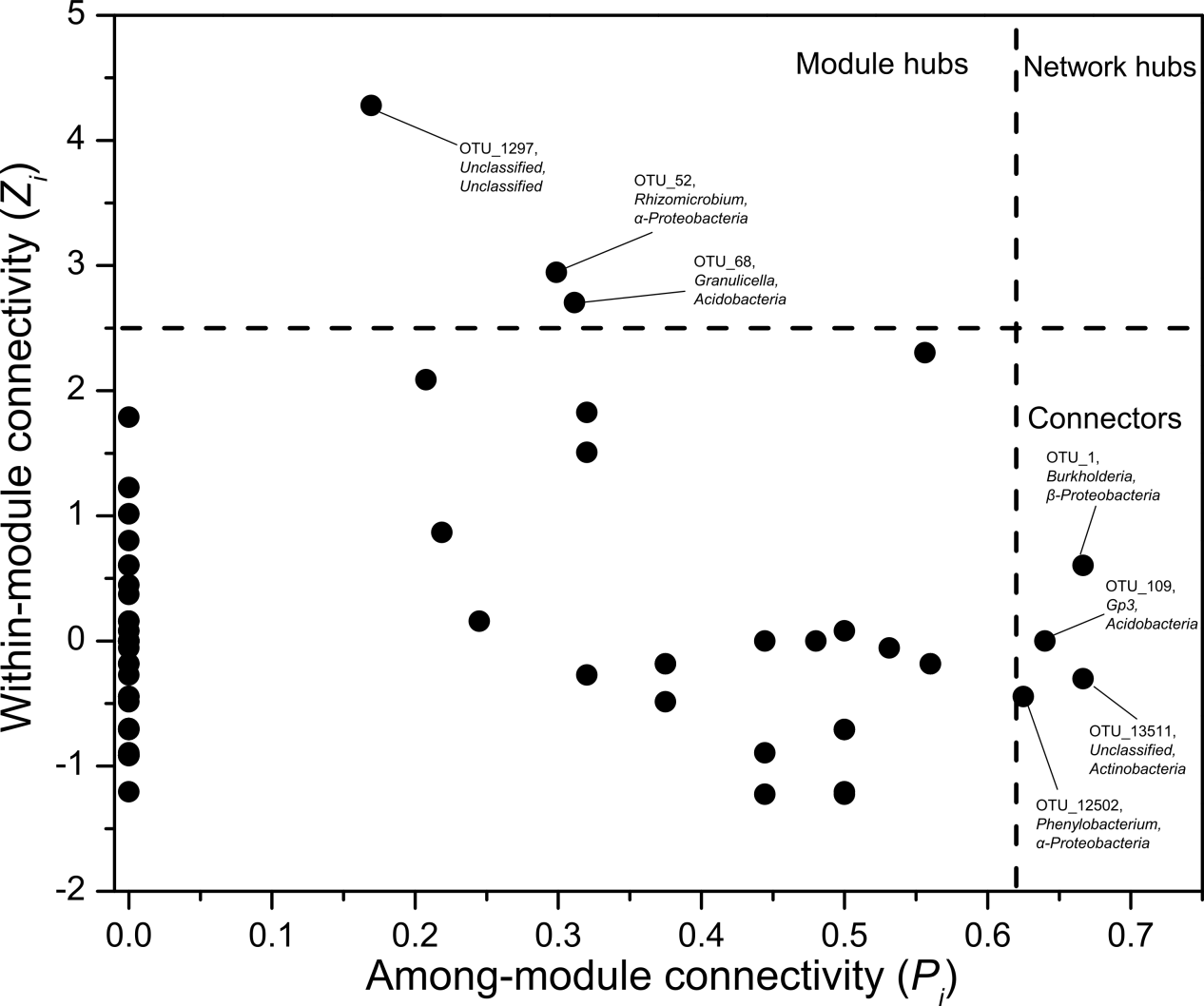


**Supplementary Figure 4.** *Z-P* plot showing the distribution of OTUs based on their topological roles. Each symbol represents an OTU. The topological role of each OTU was determined according to the scatter plot of within-module connectivity (*Z_i_*) and among-module connectivity (*P*). The module hubs and connectors are labeled with OTU numbers.


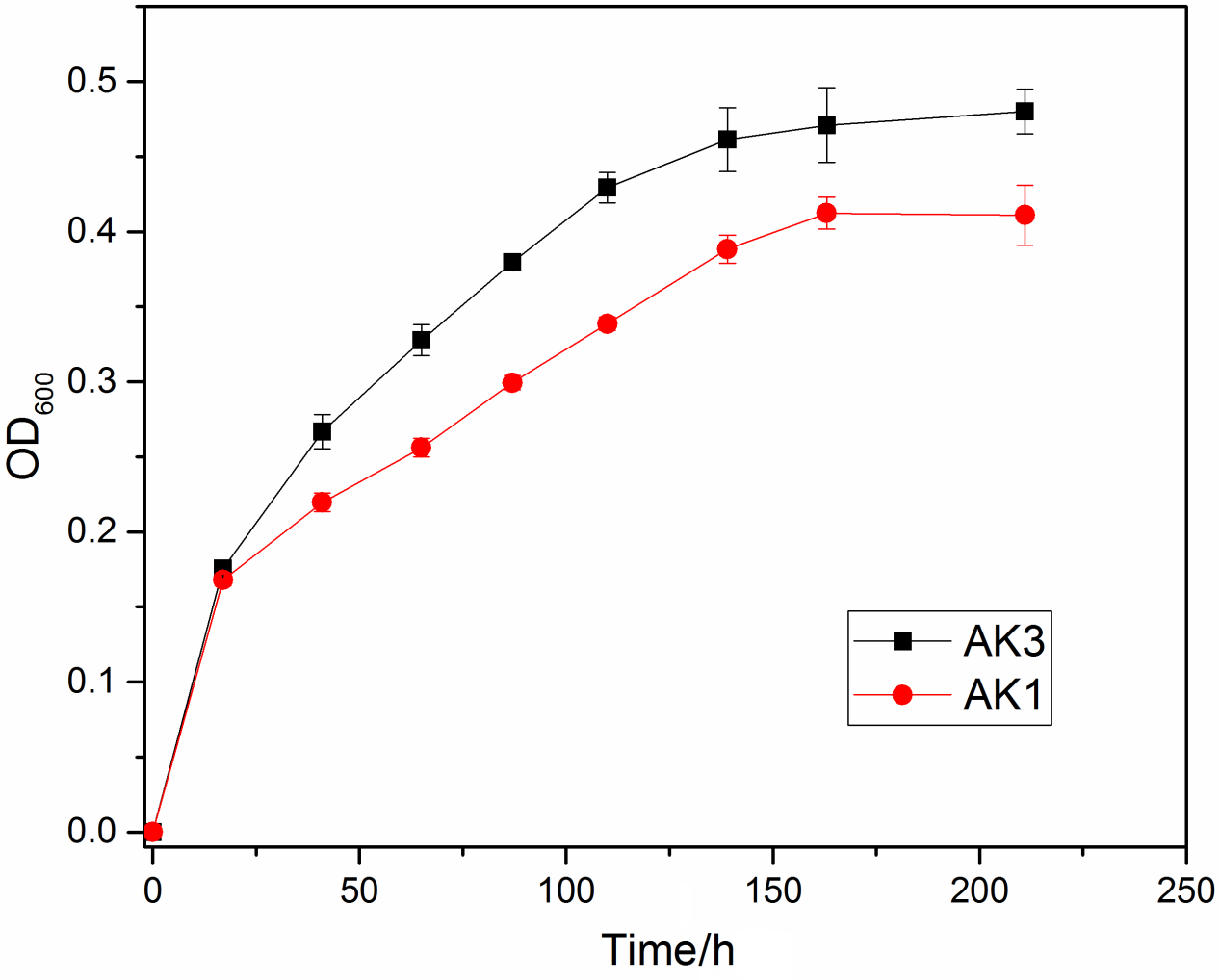


**Supplementary Figure 5.** Growth curves of *Burkholderia* strains AK3 and AK1 in defined BMM medium with alkaline lignin as the sole C substrate (*n*=3). Data are shown as mean ± standard error.


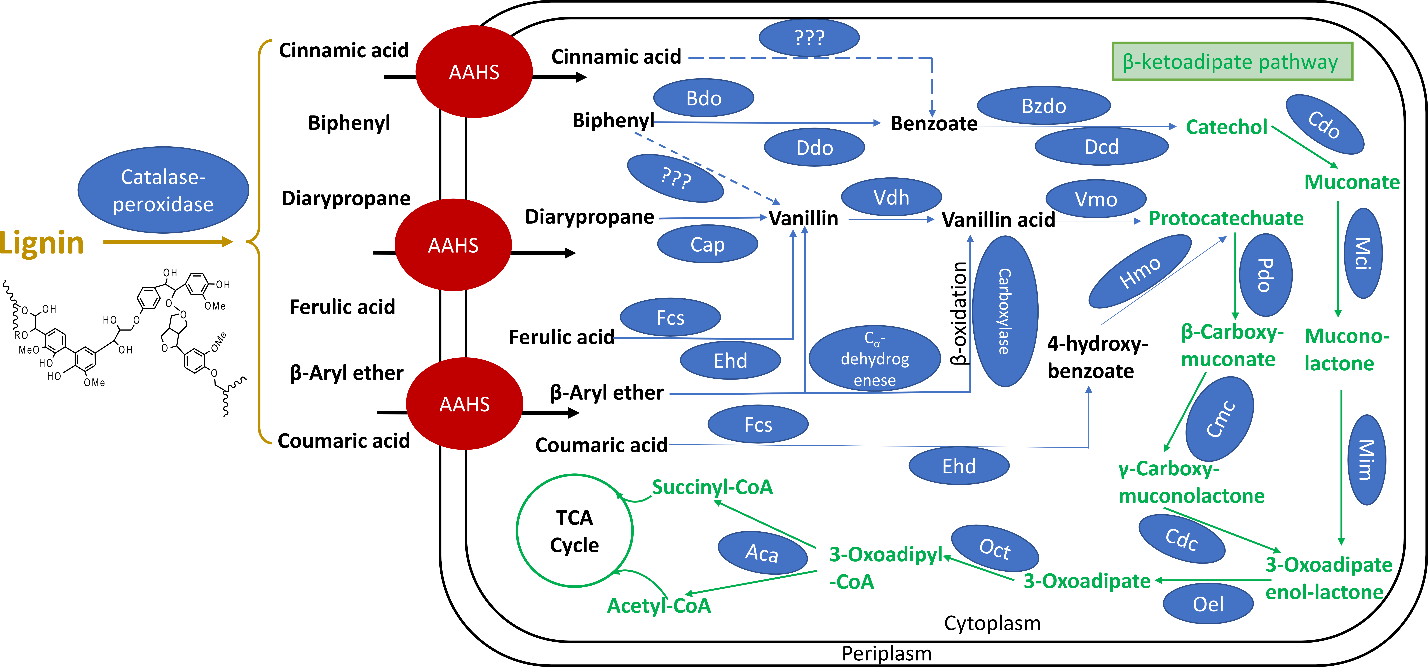


**Supplementary Figure 6.** Predicted lignin decomposition pathway in *Burkholderia* AK1 and AK3. The lignin is initially oxidized into small compounds (β-Aryl ether, Biphenyl, Diarylpropane, Ferulic acid, coumaric acid and Cinnamic acid) by catalase-peroxidases, and those small molecular compounds may be transported by Aromatic acid:H^+^ symporter (AAHS) family transporters into cell. Cinnamic acid and Biphenyl can be oxidized to benzoate and furthered oxidized to Catechol; Diarylpropane, ferulic acid and β-Aryl ether can be oxidized to vanillin firstly and then further oxidized to protocatechuate. Coumaric acid can be oxidized to 4-hydroxy-benzoate and then further oxidized to protocatechuate. The catechol and protocatechuate can be oxidized to succinyl-CoA and acetyl-CoA by β-ketoadipate pathway (Green) and enter into TCA cycle. Enzymes potentially involve in the lignin deploymerization are indicated: Cap, Catalase-peroxidase; Bdo , biphenyl dioxygenase; Ddo, 2,3-dihydroxybiphenyl dioxygenase; Fcs, feruloyl-CoA synthase; Ehd, enoyl-CoA hydratase; Vdh, Vanillin dehydrogenase; Vmo, vanillate monooxygenase; Bzdo, benzoate/toluate 1,2-dioxygenase; Dcd, dihydroxycyclohexadiene carboxylate dehydrogenase; Hmo, hydroxybenzoate 3-monooxygenase; Cdo, catechol 1,2-dioxygenase; pdo, protocatechuate 3,4-dioxygenase; Mci, muconate cycloisomerase; Cmc, 3-carboxy-cis,cis-muconate cycloisomerase; Mim, muconolactone D-isomerase; Cdc, 4-carboxymuconolactone decarboxylase; Oel, 3-oxoadipate enol-lactonase.


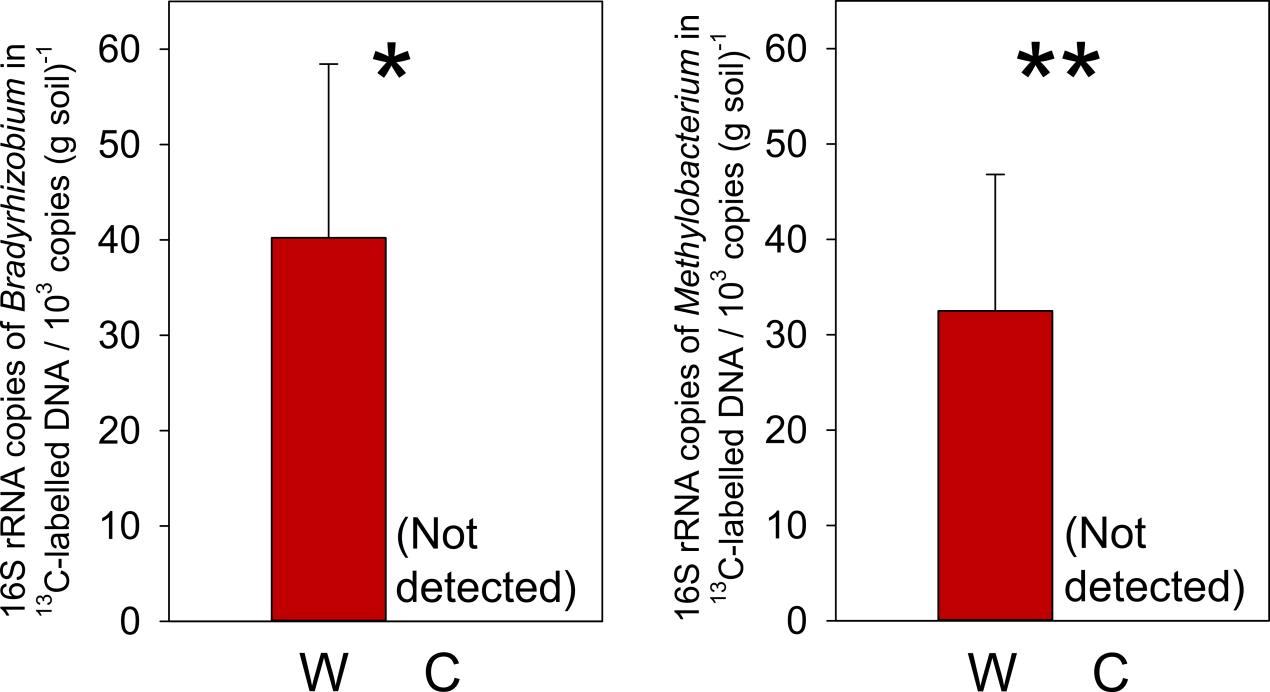


**Supplementary Figure 7.** (a) The number of ^13^C-labelled 16S rRNA gene copies of *Bradyrhizobium*; (b) The number of ^13^C-labelled 16S rRNA gene copies of *Methylobacterium*. Significance is indicated by *, 0.01<*P*≤0.05 as determined by two-tailed *t*-test (*n*=3 biological replicates). Data are shown as mean ± standard error.


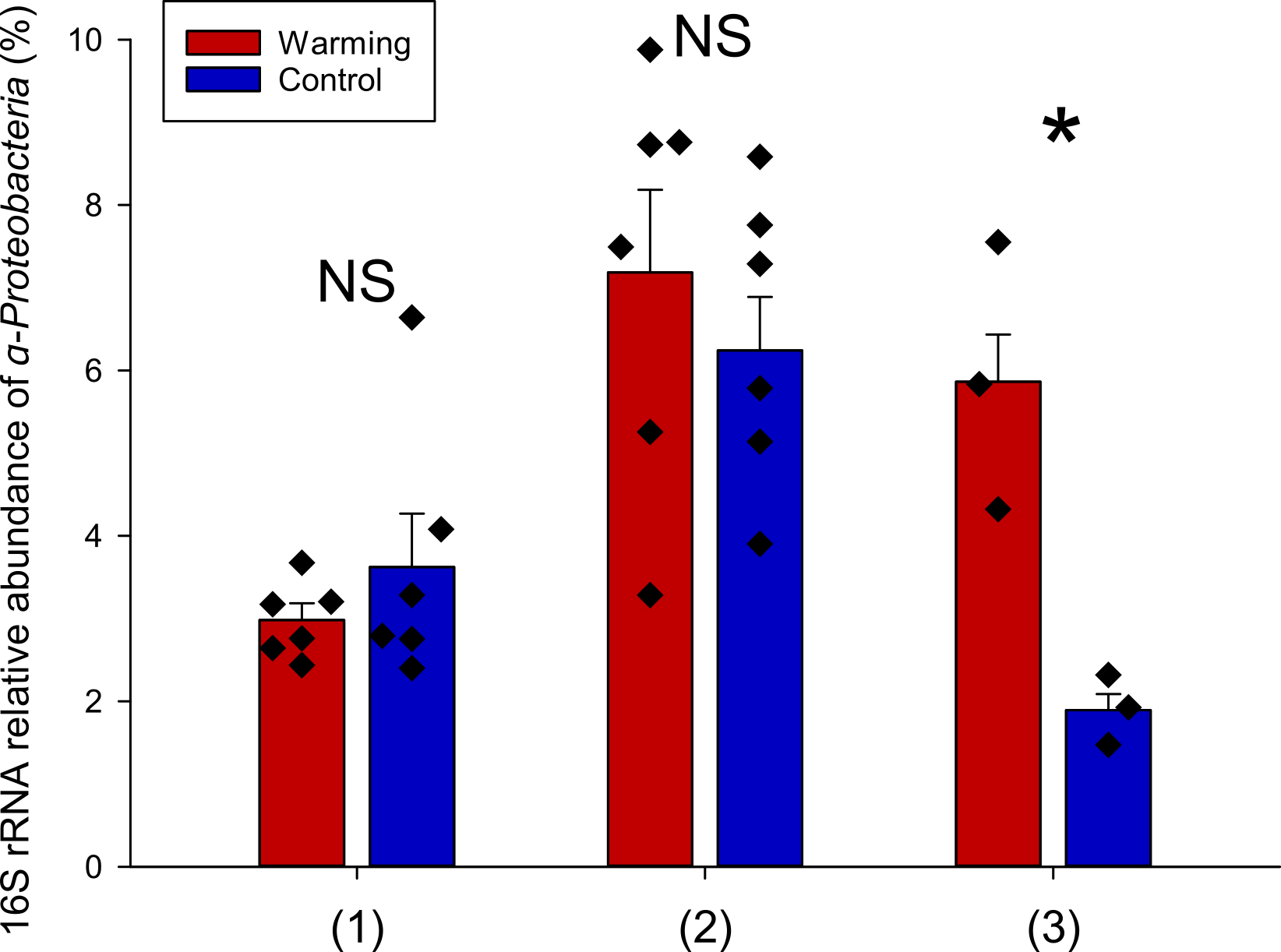


**Supplementary Figure 8.** Relative abundances of active *α-Proteobacteria* at different time points during the experiment, as revealed by 16S rRNA gene amplicon sequencing. The X-axis shows samples of different time points of: (1), before the 975-day lab incubation; (2), after the 975-day lab incubation; (3), after the 6-day SIP incubation. Significance is indicated by *, 0.01< *P* ≤0.05 and NS, *P* > 0.05 as determined by a two-tailed *t*-test (*n* = 6 before and after 975-day laboratory incubation, and *n* = 3 after the 6-day SIP incubation). Data are shown as mean ± standard error.
